# Using Visual Trepan to Treat Single Segment Ossification of the Ligamentum Flavum Under Endoscopy

**DOI:** 10.1111/os.12538

**Published:** 2019-10-29

**Authors:** Wei Zhao, Sen Yang, Wen‐bo Diao, Ming Yan, Wen‐jie Wu, Fei Luo

**Affiliations:** ^1^ Department of Orthopaedics Liu Dong Branch of Liuzhou Maternal and Child Health‐Care Hospital Liuzhou China; ^2^ Department of Orthopaedics Hospital, Third Military Medical University Chongqing China; ^3^ Zhoukou Xiehe Orthopaedics Hospital Henan China; ^4^ Department of Orthopaedics, Affiliated First Hospital Jilin University Jilin China

**Keywords:** Decompression, Endoscope, Ossification of ligamentum flavum, Thoracic spinal, Visual trepan

## Abstract

This article describes the trepan technique for treating single segment ossification of the ligamentum flavum (OLF) using an endoscope. OLF is the most common cause of thoracic spinal stenosis. The most common surgical procedures involve semi‐lamina or full‐lamina resection and decompression. However, considering the anatomical structure of the thoracic spinal canal and the combination of OLF, traditional surgery has higher risks, more complications, and greater technical requirements. In the past ten years, with the development of endoscopic technology, spinal endoscopy has been increasingly applied for the treatment of intervertebral disc herniation and spinal canal stenosis. The present study demonstrated the effectiveness of visual trepan decompression under spinal endoscopy used for patients with single segment OLF. This surgical procedure had many advantages, including a shorter operation time, minimal trauma, less expenditure, and better functional recovery over the conventional open surgery.

## Introduction

Ossification of the ligamentum flavum (OLF) is the most common cause of thoracic spinal stenosis^1^. Considering its complicated and atypical clinical manifestation, patients may delay treatment due to misdiagnosis, resulting in irreversible spinal cord damage and nervous lesions. At present, the thoracic posterior wall resection and decompression method is the main approach used to treat single segment OLF. The most common surgical procedures involve semi‐lamina or full‐lamina resection and decompression and have an excellent therapeutic effect^2^, ^3^. However, considering the anatomical structure of the thoracic spinal canal (narrow lumen and limited spinal cord buffer space) and the combination of OLF, the thoracic spinal canal is even narrower. Any instrument in the vertebral canal is likely to cause dura mater tears and intraoperative neurovascular injury, leading to obvious dysfunction. As a result, traditional surgery has higher risks, more complications, and greater technical requirements.

In the past ten years, with the development of endoscopic technology, spinal endoscopy has been increasingly applied for the treatment of intervertebral disc herniation and spinal canal stenosis^4^, ^5^. However, few reports have focused on using spinal endoscopy for thoracic vertebra OLF. The present study discusses the clinical efficacy of visual trepan decompression under spinal endoscopy for patients with single segment OLF. We describe the procedure of this operation in a typical case and introduce the specific use of visual trepan. In this case, we examined the safety and efficacy of this operation. We observed and compared the sensation, muscle strength, Japanese Orthopaedic Association (JOA) scores, and visual analogue scale (VAS) scores preoperation, during operation, 1‐month postoperation, and 6 months post‐operation. The operation time, time in bed, blood loss, complications, symptom relief, and postoperative neurological and functional recovery were also recorded. This study evaluates the application value of this new surgical method, and provides a reference for treatment choices for single segment OLF.

## Technique

### *Case Presentation*

**Figure 1:**
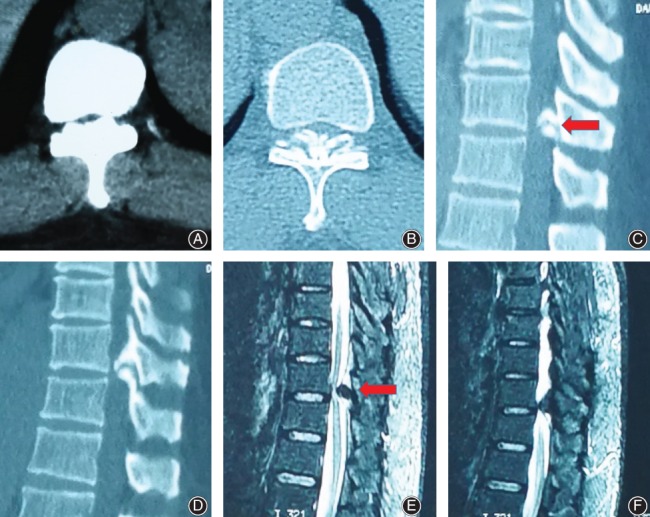
Preoperative imaging data (T_10, 11_). Thoracic CT (A, B, C, D) and MRI (E, F) suggested that the yellow ligament of the T_10, 11_ was thickened and calcified, and the spinal canal was severely narrowed in the corresponding plane.

**Figure 2:**
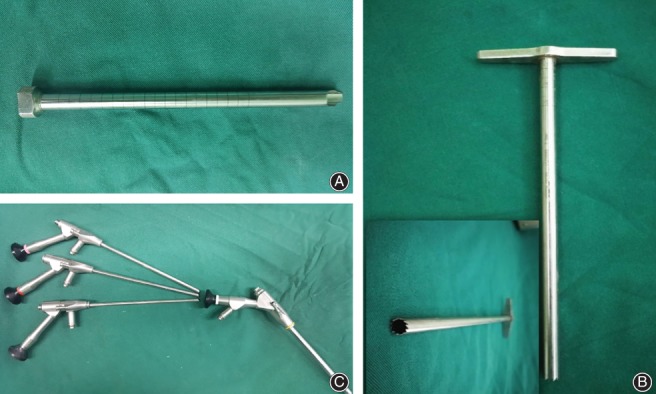
Main surgical instrument: (A) guide sleeve; (B) visual trepan; and (C) endoscope.

A 48‐year‐old male patient presented with numbness and weakness in both lower limbs for 20 days; he had experienced difficulty standing up and walking independently. Physical examination revealed tenderness in the chest and back; numbness of the skin occurred when acupuncture was applied below the left inguinal plane; hypoesthesia was observed when needling the skin in the saddle area. Muscle strength of lower limbs was grade III, and the patient had high muscle tension. Muscle strength of left and right femoral quadriceps muscle was grade III and grade IV, respectively (Table 1). The tendon reflexes of bilateral biceps and triceps humerus are normal. Ankle clonus and patellar clonus were positive, and the pathological reflex Babinski sign was positive. Thoracic CT and MRI suggested that the yellow ligament of the T_10, 11_ was thickened and calcified, and the spinal canal was severely narrowed in the corresponding plane (Fig. 1). Diagnosis on admission of this patient was T_10, 11_ segmental thoracic OLF. The operative method was thoracic canal enlargement and decompression by using visual trepan under an endoscope (Fig. 2).

**Table 1:** Clinical evaluation preoperation and postoperation

Times	Sensation (left lower limb)	Muscle strength (left lower limb)	JOA	VAS
Preoperation	Numbness	Grade III	6	5
During operation	Reduced numbness	Grade III	—	—
1 month after surgery	Slight	Grade IV	11	2
6 months after surgery	Normal	Grade IV+	14	0

JOA, Japanese Orthopaedic Association; VAS, visual analogue scale.

### *Surgery Technique*

**Figure 3:**
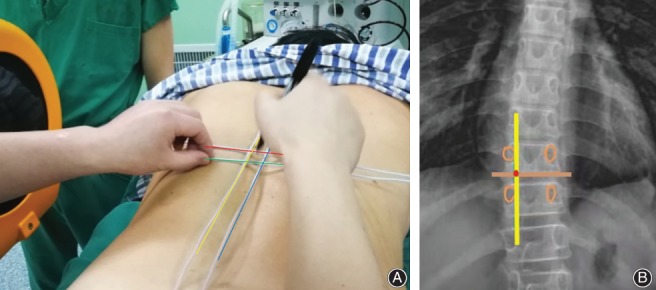
Incision center point localization. The left surgical approach was established in prone position. (A) Red line: T_10_ lower pedicle edge; green line: T_11_ upper articular process shoulder; yellow line: T_10_ pedicle inner wall; blue line: posterior median line. (B) The center of the incision is at the intersection of the yellow and orange lines (red point). The upper and lower four vertebral pedicles of the surgical segment were drawn under the X‐ray film (orange circle). The incision center was located at the intersection of the upper vertebral body lower margin line and the vertebral pedicle inner wall line.

The surgery was performed in a prone position. G‐arm positioning marked the posterior midline of the spinous process and the T_10, 11_ intervertebral space horizontal line (Fig. 3). The insertion point marker was located at the left posterior midline, approximately 3 cm away from the left midline, with the head tilted by approximately 30°.

**Figure 4:**
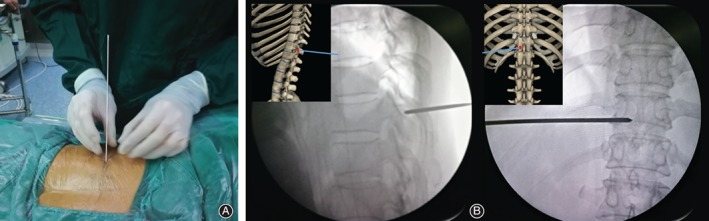
Puncture positioning. (A) 3.0 guide needle puncture; the needle point target is located at the junction of the surrounding spinous process and vertebral plate. (B) The frontal and lateral X‐ray of the position of guide needle.

**Figure 5:**
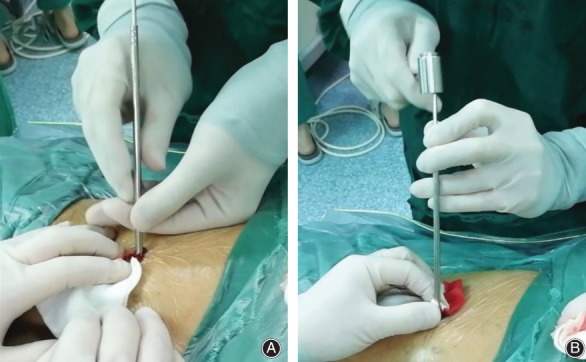
Insertion of dilation catheter and set up of working channel. (A) Make a 6‐mm long transverse incision centered on the guide needle and place a dilator catheter. (B) Tap the guide needle and pin it at the junction of the spinous process and vertebral plate.

**Figure 6:**
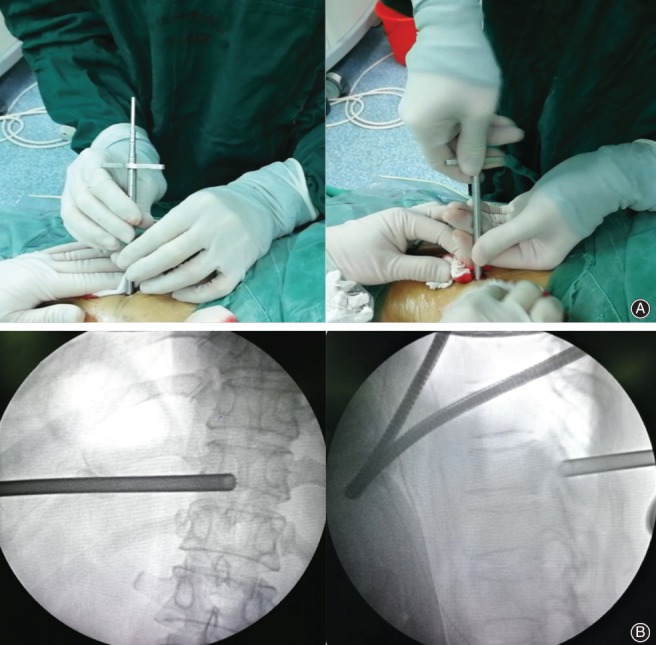
Visual trepan placement. (A) Place the visual trepan along the guide needle. (B) The frontal and lateral X‐ray of the position of visual trepan.

**Figure 7:**
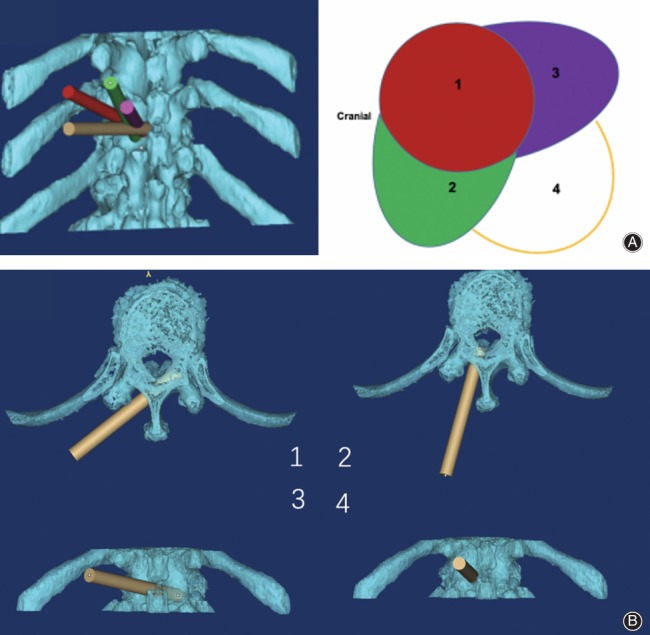
Schematic diagram of visual trepan decompression (four steps). (A) The spinous process and laminar junction were drilled to the contralateral articular process. (B) The vertebral plate and inner margin of the inferior articular process were drilled in the same side. The residual spinous process on the same side and the laminar junction to contralateral zygapophyses were drilled. The residual vertebral plate was drilled on the same side.

**Figure 8:**
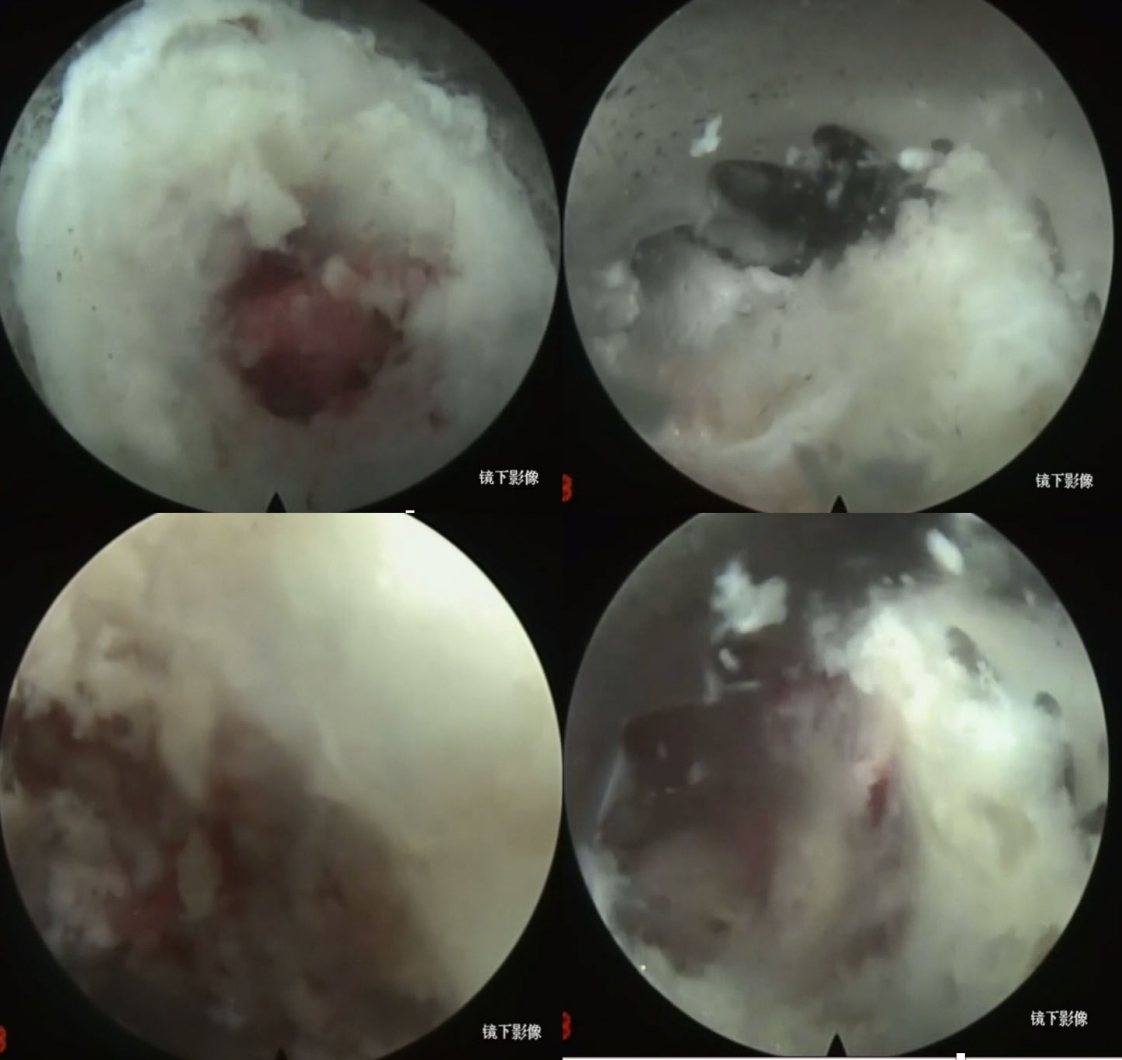
Endoscopic four‐step visualization of the images during the visible trepan surgery.

Here, 15 mL lidocaine, 10 mL ropivacaine, and 20 mL normal saline mixture was used to perform infiltration anesthesia through the skin, subcutaneous tissue, and muscle layer at the entry point of the T_9_ vertebral plate. The skin was cut to a length of 0.6 cm, and then the puncture needle was used to fix it to the T_10_ vertebral plate. The dilating catheter was installed step by step and a protective case was installed, after which all catheters were removed. The micro‐endoscope was installed and the radio‐frequency electrode was used to clean soft tissue; the superior and inferior T_10_ vertebra plate, the spinous processes of the lamina migrate, and the T_10,11_ zygapophyseal joint were visible (Figs 4, 5, 6). After the first trepan was slowly rotated into the sclerostin from upper third of the T11 hypozygal medial border vertebral plate (head inclination of 30° and backward inclination of 40°). The sclerostin showed a slight sense of loosening during the rotation of the trepanl. The right hand lightly broke the sclerostin using the trepan, and then lightly rotated the trepan to remove the bone block. Meanwhile, myodynamia changes of patients were investigated. After electrode hemostasis, the surgeon exposed part of the dural sac and the medial margin of the superior articular process of the contralateral T_11_. The annular tube, was moved to the same side, revealing a T_10_ inferior articular process. The second trepan was inserted in the superior part of the inferior articular process of T_10_ on the same side. Medial sclerostin of the inferior articular process was gently removed (1/3 horizon space was reserved in the trephine). The superior border of the spinous process on the T_10_ vertebral plate was exposed. The third trepan gently removed the spinous process basilar part and the inner plate of the contralateral vertebral plate from the upper T_10_ spinous process and vertebral plate (tail inclination approximately 10° and back inclination approximately 30°). At least 1/3 of the horizon space was reserved in the trepan. After taking out the bone block, the dural sac, the contralateral T_10_ pedicle, and the superior articular process were exposed. Finally, the annular tube was moved to the same side to expose the lower inferior articular process of the T_10_ on the same side. The lower inner margin sclerostin of the inferior articular process and the superior articular process inner margin sclerostin on the T_11_ were removed. The dural sac was explored to reveal the ideal range for the laminectomy decompression. The power system and vertebral plate rongeur under the endoscope were used to remove residual sclerostin in the inner margin of the superior and inferior articular process, sclerostin of the lower basilar part of the T_10_ spinous process, and the upper margin sclerostin of the T_11_ vertebral plate (Figs 7, 8). The ideal decompression range for up and down and both sides of the vertebral canal was explored. The dural sac was loose and had no pressure. Pulsation of endorhachis was good. There was no active bleeding in the vertebral canal. Finally, the incision was closed. The operation time was 160 min and the blood loss was 20 mL.

## Result

Postoperatively, mannitol, and hexadecadrol were used for 2–3 days. Antibiotics were used for infection prevention for 1–2 days. Meanwhile, analgesia and trophic nerve drugs were given as symptomatic treatment. The patient was bedridden for 1 day after the operation without complications, and moved with a brace on the second day after surgery.

The visual analogue scale (VAS) score was 5 and the Japanese Orthopaedic Association (JOA) score was 6 before the operation; 1 month after surgery, the VAS score was 2 and the JOA score was 11, and the muscle strength of both lower limbs was grade IV and the numbness was significantly reduced. The area of the spinal canal was 10% before the operation and nearly 100% after the operation. When the patients were followed up at 6 months after the operation, the sensation of both lower limbs had returned to normal, and the muscle strength of both lower limbs was grade IV+; the VAS score was 0 and the JOA score was 14 (Table 1).

## Discussion

Ossification of the ligamentum flavum is the primary cause of thoracic stenosis. For concealed onset and the slow development of illness, it is often ignored, meaning that it causes oppression of the nerve and spinal cord. Therefore, spinal cord blood is reduced, resulting in irreversible damage. Patients often show numbness of the lower limbs, paresthesia, and dysbasia. The thorax and abdomen have zonesthesia, and quality of life is affected. When there is a spinal cord injury, surgery is the only therapeutic option; thus, it is extremely important to diagnose and cure OLF in a timely manner.

It is difficult to achieve favorable results through conservative treatment. Operative decompression is the only effective means of treatment before irreversible damage. Posterior full‐lamina or semi‐lamina decompression is the traditional operative method for the treatment of OLF. Multiple studies have demonstrated that thoracic vertebra wall excision is the safest and most effective operative method to cure thoracic vertebra OLF^6^, ^7^, ^8^, ^9^. Wang *et al*.^7^ used the en bloc surgical method centrum posterior column to cure 18 OLF patients. It was found that after the operation, patients showed good neural functional recovery, but the amount of bleeding was greater, with an average of 691.1 ± 443.3 mL. Leakage of cerebrospinal fluid is the most common complication. Park *et al*.^8^ used vertebral plate decompression to cure 8 OLF patients. The JOA score before the operation was less than 5, but after the operation, it was improved by between 3 and 10. Back pain, zonesthesia, and paresthesia of the lower limbs were relieved to varying degrees. However, the operative procedure was needed to excise the vertebral plate and zygopophysis, peel off paravertebral muscle, excise the posterior column structure, and totally expose the intraspinal structure; thus, muscle injury risks were increased. Meanwhile, the operative incision was large, which could easily lead to poor spinal stability and induce a series of complications, including incision pain, myasthenia, and incision infection. Moreover, after removing the protective structure from the back of the nerve, the nerve and dura mater would be disturbed by the surrounding scar tissue, resulting in iatrogenic stenosis or postoperative chronic back pain. In addition, postoperative myasthenia and amyotrophy caused by paravertebral muscle might result in chronic back pain.

**Figure 9:**
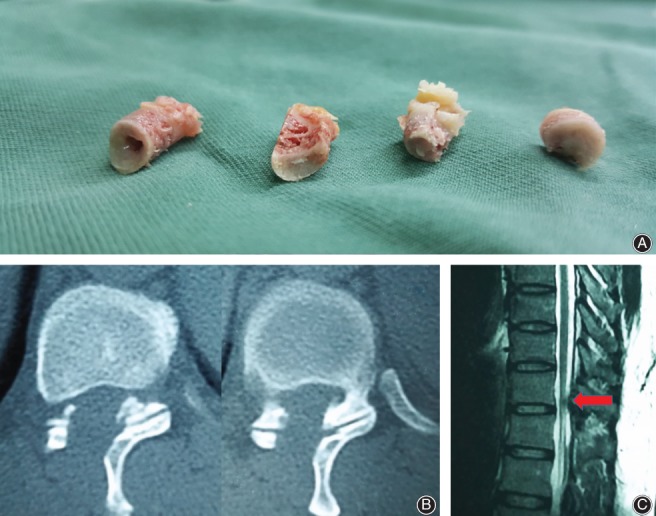
(A) Bone fragments of the ligamentum flavum were removed during surgery. (B) CT and MRI 1‐month postoperatively (T_10, 11_).

The question remains: How can the maximum operation result be gained with the minimum trauma? With the in‐depth knowledge of anatomical structure attained and the development of endoscopy, minimally invasive spinal surgery emerged at the right moment. Particularly in recent years, studies on various minimally invasive technologies have been published successively. From the traditional micro endo disc system (MED) to an intervertebral aperture mirror, and from minimally invasive spine surgery‐transforaminal lumbar interbody fusion (MISS‐TLIF) to extreme lateral interbody fusion (XLIF)/oblique lumbar interbody fusion (OLIF), various diseases requiring spinal surgery have been covered. On the basis of a preliminary study, on the philosophy of endoscope surgery, the traditional open operative steps can be completed under endoscopy to reduce operative difficulty. In this study, the combined work channel of the endoscope was used to remove OLF under an endoscope without peeling off or excising the paravertebral muscle. The work channel, with a diameter of 8.4 mm, could directly situate in the vertebral plate to protect the nervus vascularis and reduce bleeding. In addition, the 7.5‐mm visual trepan was used to replace the traditional vertebral plate rongeur, to reduce intrusion for the narrow intraspinal structure, and to effectively prevent dura mater tearing and intraoperative neurovascular injury. Other advantages include the clear surgical field, less bleeding, and fewer complications. At present, few studies in the literature have reported on the treatment of thoracic vertebra OLF under full endoscopy. Chen^9^ used percutaneous microchannel microscopic surgery to cure 68 patients with focal OLF. After surgery, the improvement in the JOA score was obvious. Recovery rates were good (up to 97.06%). The improvement rate was also increased, up to 90.86%. The procedure was performed by visual trepan under endoscopy (Fig. 9). At the 6‐month follow‐up, the JOA was obviously increased. In addition, spinal cord function was improved to some degree. Patient function recovered considerably (Table 1). Moreover, there were no relevant complications.

Relevant biomechanics and clinical studies have demonstrated that when the vertebral plate or zygopophysis incision is less than 50%, the stability of the spinal segment is not affected^10^, ^11^. Our finite element analysis (not yet published) also proves that using trepan decompression under endoscopy (four‐trepan) does not result in any obvious instability of the spine. In our opinion, this technology can be used for thoracic vertebra OLF and OPLL.

### *Core Technologies (Four‐step Operation)*

1. The spinous process and laminar junction were drilled to the contralateral articular process
2. The vertebral plate and inner margin of the inferior articular process were drilled on the same side.
3. The residual spinous process on the same side and the laminar junction to contralateral zygapophyses were drilled
4. The residual vertebral plate was drilled on the same side

#### *Highlights*

1. Visual trepan provides a new surgical method to treat OLF under an endoscope.
2. The visual trepan technique is feasable and is superiority in treating single segment OLF.
3. Visual trepan decompression has the advantages of less trauma, faster recovery, and less expenditure.
