# Erratum

**DOI:** 10.1111/os.12602

**Published:** 2020-02-19

In [1], the second affiliation was erroneously published as “Department of Orthopaedics Hospital, Third Military Medical University, Chongqing, China”.

The correct affiliation should be “Department of Orthopaedics, Southwest Hospital, Third Military Medical University, Chongqing, China.”

The publisher apologizes for the error and any convenience it may have caused.
